# An Investigation on the Clinical Features and Neurochemical Changes in Parkinson's Disease With Depression

**DOI:** 10.3389/fpsyt.2018.00723

**Published:** 2019-01-18

**Authors:** Teng-Hong Lian, Peng Guo, Li-Jun Zuo, Yang Hu, Shu-Yang Yu, Li Liu, Zhao Jin, Qiu-Jin Yu, Rui-Dan Wang, Li-Xia Li, Ying-Shan Piao, Wei Zhang

**Affiliations:** ^1^Department of Neurology, Beijing Tiantan Hospital, Capital Medical University, Beijing, China; ^2^Department of Internal Medicine, Beijing Tiantan Hospital, Capital Medical University, Beijing, China; ^3^Center for Movement Disorder, Department of Neurology, Beijing Tiantan Hospital, Capital Medical University, Beijing, China; ^4^Center for Cognitive Neurology, Department of Neurology, Beijing Tiantan Hospital, Capital Medical University, Beijing, China; ^5^China National Clinical Research Center for Neurological Diseases, Beijing, China; ^6^Key Laboratory for Neurodegenerative Disorders of the Ministry of Education, Capital Medical University, Beijing, China; ^7^Center of Parkinson's Disease, Beijing Institute for Brain Disorders, Beijing, China; ^8^Beijing Key Laboratory on Parkinson's Disease, Beijing, China

**Keywords:** depression, Parkinson's disease, risk factor, dopamine, serotonin

## Abstract

**Objective:** To investigate the clinical features and neurochemical changes in Parkinson's disease with depression (PD-D).

**Methods:** A total of 478 PD patients were divided into PD-D and PD patients without depression (PD-ND) groups according to the 24-item Hamilton Depression Rating Scale (HAMD) score. Demographic variables, motor and non-motor symptoms and activities of daily living were evaluated. The independent influencing factors of PD-D were investigated via binary logistic regression analysis. The levels of neurotransmitters in cerebrospinal fluid (CSF) were measured and their correlations with HAMD score were analyzed.

**Results:** The proportion of PD-D was 59.0%, of which 76.95, 20.92, and 2.13% had mild, moderate, and severe depression, respectively. Anxiety/somatization was the most prevalent sub-factor of HAMD in PD-D. The scores of UPDRS III, postural instability/gait difficulty (PIGD) type and the scores of 14-item Hamilton Anxiety Scale (HAMA) and 14-item Chalder Fatigue Scale (FS) were independently associated with PD-D. The levels of dopamine (DA) and 5-hydroxytryptamine (5-HT) were all significantly reduced in PD-D group compared with those in PD-ND group. HAMD scores were negatively correlated with the DA levels in CSF.

**Conclusions**: PD patients have a high proportion of depression, mainly of mild and moderate levels. The profile of depression in PD population is subtly different from that of the general population. Motor symptoms, PIGD type, anxiety and fatigue are the significant influencing factors of PD-D. Compared to 5-HT, DA may play a more important role in PD-D.

## Introduction

Parkinson's disease (PD) is a common neurodegenerative disorder with both the characteristic motor symptoms and a variety of non-motor symptoms. Depression, one of the most common non-motor symptoms of PD (1), used to be regarded as a psychological reaction to PD, and thus frequently be underestimated and undertreated. The failure to detect PD with depression (PD-D) and offer timely treatment would lead to worse outcomes for patients and caregivers (2).

Clinically, studies on PD-D have yielded conflicting results. In terms of demographic information, for example, some investigators showed that the frequency of PD-D increased with age (3), while others claimed that younger patients were more susceptible to PD-D (4). Some studies identified that being female and having a longer disease duration were the risk factors for PD-D (5), but some did not (6). From the aspect of symptoms, worse motor symptoms might be associated with PD-D (7). However, there were few studies that investigated the relationship between clinical type of PD and PD-D. For non-motor symptoms, most studies agreed that anxiety was the independent influencing factor (8), but other non-motor symptoms, for instance, fatigue, cognitive decline and sleep disturbances, had considerably varied reports (4, 9). Therefore, there is a lack of consensus on the influencing factors of PD-D. Studies consisting of large samples are necessary to explore both general and specific factors related to PD-D. In addition, there are few studies investigating the sub-factors of the 24-item Hamilton Depression Scale (HAMD) in PD-D.

The underlying mechanisms of PD-D have not been adequately clarified. Currently, PD-D is known to be of biological cause, and may be affected by multiple factors including genetic predisposition, biochemical disturbances, and psychological events (7, 10). The altered levels of neurotransmitters may serve as the biochemical basis of depression. 5-hydroxytryptamine (5-HT), dopamine (DA), and norepinephrine (NE), which were altered in the pathological process of PD, were known to play important roles in primary depression (11). Therefore, the pathophysiology of PD-D might also be related to the changes in the serotonergic, dopaminergic and noradrenergic systems (12). However, the changes in the above neurotransmitters for PD-D patients may not be the same as those in patients with idiopathic depression, which major biochemical compromise is the serotonergic system. To our best knowledge, there are limited studies on the neurotransmitters levels in cerebrospinal fluid (CSF) from PD-D patients.

In this study, PD-D was assessed using HAMD (13); motor symptoms and other non-motor symptoms were evaluated by a series of rating scales; the levels of 5-HT, DA, and NE in the CSF were measured by high performance liquid chromatography (HPLC), and their correlations with HAMD scores were analyzed. The objectives of this study were to investigate the clinical features, influencing factors and neurochemical mechanisms of PD-D.

## Methods

### Ethics Statement

This study met the guidelines of Helsinki Declaration on ethical principles for medical research involving human subjects, and the protocol was approved by the ethical review board of Beijing Tiantan Hospital. All participants signed written informed consents before they were recruited in the study.

### Participants

Total 500 PD patients were consecutively recruited from the Department of Neurology and Geriatrics of the Beijing Tiantan Hospital from November 2014 to November 2017. Patients were diagnosed with idiopathic PD based on the criteria of the Parkinson's UK Brain Bank (14).

Exclusion criteria included severe systemic diseases, such as heart failure, pulmonary disease, gastrointestinal disorders, anemia, infectious disease and chronic inflammatory disease, deep brain stimulation, and conditions that might interfere with the reliable completion of clinical assessments.

Demographic variables including sex, age, age of onset, disease duration, side of onset, education level and anti-parkinson therapy, including the types of drugs, levodopa equivalent daily dose (LEDD) and the durantion of taking medicines, etc. LEDD was calculated as previously proposed (15).

### Clinical Assessment For PD Patients

#### Depression

PD-D was diagnosed according to the diagnostic criteria for depression in PD (16). Patients with depression resulted from systemic diseases, organic mental disorders, psychoactive substances or non-addictive substances and patients on anti-depressants were excluded from analyses.

Twenty-two patients were excluded due to ineligibility. A final total of 478 PD patients were included in the research. Patients with HAMD score of ≥8 points and fulfilled the above diagnostic criteria were assigned to the PD-D group. Within the PD-D group, patients with HAMD scores of 8–19 points, 20–34 points and ≥35 points were categorized as having mild, moderate and severe depression, respectively. PD patients with HAMD scores of < 8 points were assigned to the group of PD patients without depression (PD-ND).

The 24 items of HAMD can be grouped into the following 7 factors (5): (1) anxiety/somatization (6 items: psychic anxiety, somatic anxiety, gastrointestinal symptoms, hypochondriasis, insight, and general symptoms); (2) weight loss (1 item); (3) cognitive disturbances (6 items: self-guilt, suicide, agitation, depersonalization and derealization, paranoid, and obsessive-compulsive symptom); (4) circadian fluctuations (1 item); (5) retardation symptoms (4 items: depression, work and interests, retardation, and sexual symptoms); (6) sleep disturbances (3 items: difficulty falling asleep, superficial sleep and early awakening); (7) hopelessness symptoms (3 items: helplessness, hopelessness and worthlessness).

#### Motor Function, Non-motor Symptoms, and Activities of Daily Living

The severity of PD was evaluated according to the Hoehn and Yahr (H-Y) stage. The motor symptoms were assessed using the Unified Parkinson's Disease Rating Scale (UPDRS) III. PD patients were divided into three phenotypes according to the clinical phenotype classification by Jankovic (17): tremor-dominant (TD) subtype, postural instability and gait difficulty (PIGD) subtype and mixed subtype. Items 16, 20, and 21 of the UPDRS were for tremor symptoms; items 13, 14, 15, 29, and 30 of UPDRS were for PIGD symptoms. The phenotype was defined by the ratio of the mean tremor score to PIGD score. TD subtype was defined as the ratio ≥1.5, PIGD subtype as the ratio ≤ 1.0 and mixed subtype as the ratio between 1.0 and 1.5.

A variety of non-motor symptoms were assessed using the following scales: the Montreal Cognitive Assessment Scale (MoCA) (18) for cognitive impairment, the 14-item Hamilton Anxiety Scale (HAMA) (19) for anxiety, the 14-item Chalder Fatigue Scale (FS) (20) for fatigue, the Rapid Eye Movement Sleep Behavior Disorder (RBD) Screening Questionnaire (RBDSQ) (21) for RBD, the Scale for Outcomes in PD for Autonomic Symptoms (SCOPA-AUT) (22) for autonomic dysfunction, and the Restless Legs Syndrome (RLS) Severity Rating Scale (RLSRS) (23) for RLS.

Activities of daily living (ADL) were evaluated by ADL Scale.

### Measurements of Neurotransmitters in CSF From PD Patients

Anti-parkinsonian drugs were withdrawn for 12–14 h if the patients' condition allowed and longer time was considered unethical by our ethical committee. For medical washout period should be three times of the t1/2 of the medicine, the subjects taking drugs with long t1/2, including controlled release Sinemet (Sinement CR), Pramipexole and controlled release Piribedil (Piribedil CR) were excluded in the analysis of neurotransmitters. Under fasting condition, 5 ml CSF was taken in a polypropylene tube through lumbar puncture, between 7 and 9 a.m. CSF samples were centrifuged immediately at 3,000 rpm at 4°C. Then, approximately 0.5 ml volume of CSF was aliquoted into separate Nunc cryotubes and kept frozen at −80°C until usage in the assays.

The levels of neurotransmitters, including DA, 5-HT, and NE in CSF from PD patients were measured by HPLC (24). LC-MS-MS 6410 chromatograph and Phenomenex 150^*^2 mm and 150^*^3 mm chromatographic columns were from the Agilent Company (California, USA), and the standard sample was from Sigma Company (California, USA). In total PD patients, 89 patients agreed lumbar puncture and after excluding patients used drugs with long T1/2, 76 patients' neurotransmitters in CSF were analyzed.

### Statistical Analyses

Statistical analyses were performed using SPSS Statistics 20.0 (IBM Corporation, 220 New York, USA). *P*-value of less than an alpha level of 0.05 was defined as statistically significant.

Demographic information, motor and non-motor symptoms and ADL scores between PD-D and PD-ND group were compared. Continuous variables, if normally distributed, were presented as means ± SDs and the 2 groups were compared using 2-tailed *t*-test; if not normally distributed, the data were presented as median (quartile) and compared using non-parametric test. Discrete variables were compared using Chi-square test.

Binary logistic regression analysis was used to investigate the independent influencing factors of PD-D. The covariates with statistical differences in single-factor analysis were divided into a multivariate model; Backward elimination was applied to remove non-significant variables. Influencing factors were presented as odd ratios (OR) with 95% confidence intervals (CI).

Three kinds of neurotransmitters levels of CSF between PD-D and PD-ND groups were compared. Bonferroni correction was made and P was reduced to 0.017 (0.05/n = 0.05/3 ≈ 0.017). Pearson correlation analyses were conducted between HAMD scores and neurotransmitters levels in CSF in the PD group. Binary logistic regression analysis was used. The covariates contained the neurotransmitters which had statistical differences in single-factor analysis and the independent influencing factors of PD-D in the above mentioned binary logistic regression analysis; Backward elimination was applied to remove non-significant variables.

## Results

### Frequency of Depression of PD

Among 478 PD patients, 282 (59.00%) were diagnosed with PD-D: of which, 76.95% (217/282 cases) had mild depression, 20.92% (59/282 cases) had moderate depression, and 2.13% (6/282 cases) had severe depression. The remaining 196 cases (41.00%) were in the PD-ND group.

### Assessment of Each Sub-factor of HAMD for PD-D and PD-ND Groups

The assessment of each sub-factor of HAMD was shown in Table 1. Anxiety/somatization was the most common sub-factor reported in PD-D group at 99.30%, followed by retardation symptoms (97.51%) and hopelessness symptoms (89.71%). Anxiety/somatization also occurred in the largest frequency of PD-ND patients, but compared with those of PD-D, the frequency of each sub-factor of HAMD was much lower.

**Table 1:** Assessment of each sub-factor of HAMD for PD-D and PD-ND groups.

	**Frequency (*****n*****, %)**		**Score (mean ±*****SD*****)**		**Range of the score (min–max)**	
	**PD-ND**	**PD-D**	**PD-ND**	**PD-D**	**PD-ND**	**PD-D**
Anxiety/somatization	107 (54.60%)	280 (99.30%)	1.08 ± 1.21	4.64 ± 2.48	0–5	0–13
Retardation symptoms	93 (47.40%)	275 (97.51%)	0.69 ± 0.88	3.53 ± 1.86	0–4	0–12
Hopelessness symptoms	89 (45.40%)	253 (89.71%)	0.69 ± 0.90	2.76 ± 1.89	0–4	0–10
Sleep disturbances	60 (30.60%)	211 (74.82%)	0.51 ± 0.96	2.30 ± 1.84	0–5	0–6
Cognitive disturbances	28 (14.30%)	200 (70.92%)	0.14 ± 0.35	1.79 ± 1.95	0–1	0–10
Weight loss	28 (9.20%)	100 (35.46%)	0.12 ± 0.41	0.49 ± 0.73	0–3	0–3
Circadian fluctuations	12 (6.10%)	97(34.40%)	0.10 ± 0.41	0.63 ± 0.97	0–4	0–4

### Demographic Variables, Motor Function, Non-motor Symptoms, and ADL of PD-D and PD-ND Groups

Demographic variables, motor function, non-motor symptoms and ADL scores were compared between PD-D and PD-ND groups in Table 2.

**Table 2:** Demographic variables, motor function, non-motor symptoms, and ADL of PD-D and PD-ND groups.

	**PD-ND group (196 cases)**	**PD-D group (282 cases)**	***P***
**DEMOGRAPHIC INFORMATION**			
Female (*n*, %)	87 (44.39%)	150 (53.19%)	0.058
Age (year, $\bar{X}\pm s$)	63.00 (56.00–70.00)	61.50 (55.75–69.00)	0.278
Age of onset (year, $\bar{X}\pm s$)	59.87 ± 11.16	57.51 ± 10.89	0.024^*^
Disease duration [year, Median (Q1-Q3)]	2.00 (1.00–4.00)	3.00 (1.00–5.00)	<0.001^**^
Low education level (<9 years) (*n*, %)	93 (47.45%)	176 (62.41%)	<0.001^**^
Left side of onset (n, %)	82 (41.84%)	132 (46.81%)	0.282
**ANTI-PARKINSON DRUG**			
LEDD [mg, M (Q1-Q3)]	0.75 (0, 6.90)	1.16 (0, 3.00)	0.341
**Kinds of Anti-parkinson drug**			
Madopar (*n*, %)	85 (43.37%)	144 (51.06%)	0.098
Sinemet CR (*n*, %)	12 (6.12%)	20 (7.09%)	0.677
Entacapone (*n*, %)	7 (3.57%)	19 (6.74%)	0.133
Pramipexole (*n*, %)	45 (22.96%)	48 (17.02%)	0.107
Piribedil CR (*n*, %)	36 (18.37%)	49 (17.38%)	0.780
Selegiline (*n*, %)	4 (2.04%)	4 (1.42%)	0.722
Antane (*n*, %)	9 (4.59%)	23 (8.16%)	0.125
**Duration of anti-PD therapy**			
≤ 1 year	61 (31.12%)	82 (29.08%)	0.059
>1– ≤ 3years	84 (42.86%)	96 (34.04%)	
>3– ≤ 7years	35 (17.86%)	64 (22.70%)	
>7years	16 (8.16%)	40 (14.18%)	
**MOTOR FUNCTION**			
**H-Y stage (*****n*****, %)**			
Early stage (stage 1–2.5)	179 (91.33%)	224 (79.43%)	<0.001^**^
Advanced score (stage 3–5)	17 (8.67%)	58 (20.57%)	
Total UPDRS III [point, Median (Q1-Q3)]	18.00 (12.00–27.00)	28.00 (19.00–40.00)	<0.001^**^
Tremor	3.00 (2.00–6.00)	5.00 (2.00–8.00)	
Rigidity	3.00 (1.00–5.00)	5.00 (2.00–9.00)	
Bradykinesia	7.00 (5.00–11.00)	11.00 (7.00–16.00)	
Postural instability/gait difficulty	3.00 (2.00–4.00)	4.00 (3.00–6.00)	
**CLINICAL TYPE (*****n*****, %)**			
TD subtype	67 (35.40)	6 (2.70)	<0.001^**^
PIGD subtype	106 (52.40)	276 (97.30)	
Mixed subtype	25 (12.20)	0 (0.00)	
**NON-MOTOR SYMPTOMS**			
MoCA [point, Median (Q1-Q3)]	23.00 (19.00–27.00)	21.00 (16.00–25.00)	<0.001^**^
HAMA [point, Median (Q1-Q3)]	3.00 (1.00–5.00)	12.00 (8.00–17.75)	<0.001^**^
FS [point, Median (Q1-Q3)]	7.00 (4.00–10.00)	10.00 (8.00–12.00)	<0.001^**^
RBDSQ [points, Median (Q1-Q3)]	1.00 (0.00–4.00)	3.00 (1.00–7.00)	<0.001^**^
SCOPA-AUT [points, Median (Q1-Q3)]	33.00 (29.00–37.00)	36.00 (32.00–43.00)	<0.001^**^
RLSRS [point, Median (Q1-Q3)]	0.00 (0.00–6.00)	0.00 (0.00–18.00)	<0.001^**^
**ADL**			
ADL [point, Median (Q1-Q3)]	22.00 (20.00–30.00)	32.50 (23.00–42.25)	<0.001^**^

* P < 0.05;

** *P < 0.01. LEDD, levodopa equivalent daily dose; Sinemet CR, controlled release Sinemet; Piribedil C, controlled release Piribedil; UPDRS III, Unified Parkinson's Disease Rating Scale III; TD, tremor-dominant; PIGD, postural instability/gait difficulty; MoCA, Montreal Cognitive Assessment Scale; HAMA, the 14-item Hamilton Anxiety Scale; FS, the 14-item Chalder Fatigue Scale; RBDSQ, the Rapid Eye Movement Sleep Behavior Disorder Screening Questionnaire; SCOPA-AUT, the Scale for Outcomes in PD for Autonomic Symptoms; RLSRS, Restless Legs Syndrome Severity Rating Scale*.

Compared with the PD-ND group, the PD-D group showed a significantly earlier age of onset, remarkably longer disease duration, and significantly lower education level. There was no significant difference in the other demographic variables, including sex, age, side of onset and the condition usage of the anti-Parkinson drugs between the two groups.

The PD-D group had a significantly advanced H-Y stage, significantly increased UPDRS III score and higher proportion of the PIGD subtype.

The PD-D group scored significantly lower MoCA score and higher scores of HAMA, FS, RBDSQ, SCOPA-AUT, and RLSRS scales when compared with the PD-ND group, demonstrating that the PD-D group had more severe cognitive impairment, anxiety, fatigue, RBD, autonomic dysfunction, and RLS.

Compared with the PD-ND group, the score of ADL scale in the PD-D group was significantly decreased, suggesting that the PD-D group had significantly poor ADL.

### Factors Associated With PD-D

Binary logistic regression analysis was performed using the above variables that found statistical differences between PD-D and PD-ND groups. The results showed that the scores of UPDRS III, PIGD type, and the scores of HAMA and FS were independently associated with PD-D (Table 3).

**Table 3:** Logistic regression analysis of factors associated with PD-D.

	**B**	**OR**	**95% CI**	***P***
Age of onset	−0.033	0.967	0.935–1.000	0.052
UPDRS III score	0.049	1.050	1.020–1.082	0.001^*^
PIGD type	−3.611	0.027	0.007–0.106	<0.001^**^
HAMA score	0.285	1.330	1.216–1.454	<0.001^**^
FS score	0.111	1.117	1.005–1.241	0.040^*^
RBDSQ score	0.078	1.081	0.988–1.241	0.090
Constant number	−1.611	0.200		0.152

* *P < 0.05*,

** *P < 0.01. UPDRS III, Unified Parkinson's Disease Rating Scale III; PIGD, postural instability/gait difficulty; HAMA, the 14-item Hamilton Anxiety Scale; FS, the 14-item Chalder Fatigue Scale; RBDSQ, the Rapid Eye Movement Sleep Behavior Disorder Screening Questionnaire; B, regression coefficient or intercept; OR, odds ratio; CI, confidence interval*.

### Levels of Neurotransmitters in CSF From PD-D and PD-ND Groups

The levels of DA, 5-HT, and NE in CSF from the PD-ND and PD-D groups were compared (Table 4). In the PD-D group, the levels of DA and 5-HT were all reduced compared with those in the PD-ND group. However, only DA level showed a significant difference after Bonferroni correction.

**Table 4:** Levels of neurotransmitters in CSF from PD-D and PD-ND groups.

	**PD-ND group (24 cases)**	**PD-D group(52 cases)**	***P***
DA (fg/mL, $\bar{X}\pm s$)	8.042 ± 2.423	6.025 ± 2.214	0.001^*^
5-HT (fg/mL, $\bar{X}\pm s$)	19.221 ± 7.358	14.796 ± 8.365	0.029
NE (fg/mL, $\bar{X}\pm s$)	483.004 ± 140.534	496.229 ± 125.904	0.683

* *P < 0.017. DA, dopamine; 5-HT,5-hydroxytryptamine; NE, norepinephrine*.

The correlational analyses between HAMD scores and neurotransmitters levels in CSF from PD patients were further conducted (Table 5). It was found that the HAMD scores had a negative correlation with DA level in CSF (*r* = −0.278, *P* < 0.05). However, no significant relationship between HAMD scores and the levels of 5-HT and NE in CSF was detected.

**Table 5:** Correlation of HAMD scores with the levels of neurotransmitters in CSF from PD patients.

	***R***	***P***
DA (fg/ml)	−0.278	0.015^*^
5-HT (fg/ml)	−0.22	0.056
NE (fg/ml)	0.056	0.63

* *P < 0.017. DA, dopamine; 5-HT, 5-hydroxytryptamine; NE, norepinephrine*.

Binary logistic regression analysis was performed to investigate the relationship of PD-D and DA levels in CSF. The covariates contained the levels of DA in CSF and the scores of UPDRS III, PIGD type and the scores of HAMA and FS (Table 6). The results showed that the DA levels significantly negatively correlated with PD-D.

**Table 6:** Logistic regression analysis of the relationship of DA in CSF and PD-D.

	**B**	**OR**	**95% CI**	***P***
UPDRS III score	0.03	1.031	0.949–1.112	0.460
PIGD type	−2.628	0.072	0.007–1.124	0.042^*^
HAMA score	0.822	2.275	1.211–3.846	0.008^*^
DA	−1.012	0.364	0.172–0.816	0.014^*^
Constant number	3.679	39.587		0.154

* *P < 0.05. UPDRS III, Unified Parkinson's Disease Rating Scale III; PIGD, postural instability/gait difficulty; HAMA, the 14-item Hamilton Anxiety Scale; DA, dopamine; B, regression coefficient or intercept; OR, odds ratio; CI, confidence interval*.

## Discussion

Though PD was previously characterized by the classical motor symptoms, recent studies have suggested that non-motor symptoms play significant roles in the deterioration of the quality of life for PD patients (25, 26). As one of the mood disorders, depression is often covered by motor symptoms and fails to present as the chief complaint. It is important to diagnose PD-D, because it is one of the main determinants of quality of life for PD patients and lack of PD-D diagnosis results in heavy burden to the families of patients (27, 28).

The prevalence of PD-D differs greatly across studies, ranging from 2.7 to 90%, and around 35% of PD patients presented significant depressive symptoms clinically (29), which was much higher than that in general population (30). The variation might be resulted from different diagnostic criteria, rating scales, sample sizes, and study population (31). In the current study, a large sample containing 478 Chinese PD patients was established, of which, 59% of the population suffered from depression. Analysis of the severity of depression in PD patients indicated that mild, moderate, and severe depression accounted for 76.95, 20.92, and 2.13%, respectively, implying that PD-D was featured by the mild and moderate depression (9). Clinical trials have shown that mild depressive symptoms are a variable process and may result in remission or take a turn for more severe and persistent symptoms over time (32).

Previous studies rarely investigated the 7 sub-factors of HAMD-24 items in detail. In this investigation, the results showed that PD-D patients had the highest frequency of anxiety/somatization, which was followed by the symptoms of retardation and hopelessness. The cognitive disturbances, which contains suicide, had the fifth frequency. This suggested that the profile of depression in PD population was subtly different from that of the general population which showed a high rate of suicide (33).

In this study, the binary logistic regression analysis showed that the UPDRS III score, PIGD type, the scores of HAMA and FS were the significant influencing factors of PD-D. Motor symptoms were significantly evidently different between PD-D and PD-ND groups. Some earlier studies failed to find a significant relationship between motor symptoms and PD-D (9, 34); the inconsistency may be caused by patients' stronger perception of depression than actual disability in “on” phase. Worse motor symptoms may aggravate patients' psychological and physical burden, which would increase the sense of guilt and despair. Besides, our study found that PIGD type was independently associated with PD-D. Accordingly, good management of motor symptoms is important for PD-D.

In our previous study (35), PIGD patients had more severe or faster neurodegeneration than TD group. In addition, PIGD severity might be related to the depletion of homovanillic acid, one of the metabolites of DA, in CSF (35). Moreover, in the current study, the decrease of DA might correlate with PD-D. Therefore, the PIGD group might be vulnerable to PD-D. Much effort should be paid to PD patients with PIGD type to mitigate depression.

The comorbidity of depression and anxiety in PD patients was as high as 14–50% (36, 37). In this study, the logistic regression analysis demonstrated that anxiety was one of the risk factors of PD-D. Anxiety and depression in PD patients often exist together, suggesting that they may have a common biochemical basis. They may both be related to the extensive serotonergic alteration and a more limited dopaminergic breakdown (38). Moreover, increased levels of inflammatory markers in CSF were strongly associated with depression, anxiety and other non-motor symptoms of PD (39). Nonetheless, research suggests that the comorbidity of depression and anxiety might be resulted from the superposition of different pathophysiological mechanisms of anxiety and depression rather than the common mechanism (37).

Fatigue was previously considered as a manifestation of PD-D, but in recent years, it has been recognized as a non-motor symptom independent of depression. A previous study showed that fatigue was correlated with depression (40). We further demonstrated that fatigue was one of the significant influencing factors of depression in PD patients. Our previous studies found that the decrease of serotonin dysfunction might be correlated with fatigue in PD patients (24). There was much interest in whether PD-D and fatigue shared the similar pathophysiologic mechanisms, for example, serotonergic dysfunction (41, 42). The underlying mechanism needs further investigation.

The research on the mechanisms of PD-D has not reached consensus. Studies showed that other chronic diseases, which also resulted in movement disorders, had lower incidences of depression than PD (28). What's more, depressive symptoms could appear before motor symptoms in PD patients (43). Therefore, as endogenous depression, PD-D is likely related to the characteristic pathology of PD. Theoretically, according to Braak stage of PD pathology (44), Lewy bodies deposit in the raphe nuclei, locus caeruleus, substantia nigra, and ventral tegmental area, and cause progressive loss of neurons and subsequent depletion of several neurotransmitters, including 5-HT, NE, and DA, etc.

At Braak stage II, the serotonergic neurons are affected. A study reported that PD-D patients had reduced levels of 5-hydroxyindole acetic acid (5-HIAA, a metabolite of 5-HT) in CSF than PD-ND patients (45, 46). However, Olivola and investigators failed to find the changes of 5-HT in the CSF of PD-D patients (47). These studies, including our study, showed the lack of correlation between depression and 5-HT or 5-HIAA in CSF. However, a positron emission tomography (PET) imaging study indicated a close correlation between PD-D and 5-HT transporter (48), although other similar studies failed to display consistent results (49, 50). Although there are many plausible theories explaining that deficits of the monoaminergic neurotransmitters are related to depression (51), the roles of serotonergic system on PD-D are less certain in human studies.

The level of DA is increasingly recognized as an important indicator for serious problems in PD-D; this is supported by pathological, experimental, and neuroimaging studies (52). The DA transporter availability was also proven to be reduced in PD-D in several PET studies (12, 53, 54). As a DA receptor agonist, pramipexole (0.125–1.0 mg three times per day) could improve the depressive symptoms in PD patients, and 80% of the improvements was caused by a direct effect of treatment on depressive symptoms while 20% worked through the alleviation of motor symptoms (55). Pramipexole could upregulate the expression of dopamine receptor D3 (56). The same effect was found in Ropinirole (57) and a prospective multicenter study showed that Ropinirole (a median dose of 10 mg) could improve both anxious and depressive symptoms in PD patients (58). The anti-depressant effect also was obtained in Rasagiline with higher doses than those used for the control of motor symptoms (1 mg/day) (59) and it might be related with its role for dopamine-enhancing (60). Unfortunately, none of these studies reported an association between the levels of neurotransmitters in CSF and PD-D. The results of our study supported the model that PD-D arised as a result of the dysfunction in dopaminergic pathways (61). On the other hand, DA helped to improve the motor symptoms, which was an independent influencing factor for PD-D. In this study, it showed that in PD-D patients, both the DA and 5-HT levels in CSF were decreased, however, only the level of DA in CSF was correlated with HAMD score. These results suggested that DA played a more significant role on PD-D. Therefore, it is plausible to speculate that dopaminergic-centered therapy may be much helpful for PD-D.

Most of NE neurons are distributed in the locus caeruleus. Previous investigations on the association between NE and PD-D were rather limited with relatively small sample sizes. Additionally, these studies chiefly focused on the damages of NE neurons and related innervations, and their correlation with PD-D. For example, a study observed that neuronal loss in locus caeruleus was different between PD-D and PD-ND groups (62). Another PET study using ^11^C-RTI-32 as an *in vivo* marker for both DA and NE transporter binding, revealed that PD-D might be associated with the loss of NE innervations in the limbic system (12). Although the above studies showed clues implying the potential involvement of NE in PD-D, there was no direct evidence linking NE reduction in CSF to PD-D. In this study, the NE levels in CSF did not differ significantly between PD-D and PD-ND groups, and PD-D did not correlate with NE level in CSF. Therefore, NE might not be a critical neurotransmitter for PD-D.

This study has the following limitations. CSF samples were not obtained from all PD patients enrolled in this study due to difficulties including old age, hyperosteogeny, and intolerance of holding position for lumbar puncture, etc. CSF samples from PD patients with moderate and severe depression were also relatively limited. Thus, further investigations with more CSF samples, especially from moderate and severe PD-D patients and prolonged follow-up time are much needed to support the results from the current study. What's more, as a retrospective and observational study, our study provided limited grounds for drawing definite conclusions. Longitudinal studies are required to elucidate how the depression in PD patients affects the progression and prognosis of these patients.

In summary, PD patients have a high frequency of mild and moderate depression. Motor symptoms, PIGD type, anxiety and fatigue are the significant influencing factors of PD-D. DA plays a more important role on PD-D. Results from this study provide new insights for the management of the above risk factors of PD-D and possible route for reduction of PD-D by targeting dopaminergic system.

## Author Contributions

T-HL drafting the manuscript, study design, analysis of data, accepts responsibility for conduct of research and will give final approval, acquisition of data, statistical analysis. PG study design, accepts responsibility for the conduct of research and will give final approval, acquisition of data. L-JZ, YH, S-YY, LL, ZJ, Q-JY, R-DW, L-XL, and Y-SP accepts responsibility for the conduct of research and will give final approval, acquisition of data. WZ study design, analysis of data, accepts responsibility for the conduct of research and will give final approval, acquisition of data, statistical analysis, study supervision.

### Conflict of Interest Statement

The authors declare that the research was conducted in the absence of any commercial or financial relationships that could be construed as a potential conflict of interest. The reviewer FD and handling editor declared their shared affiliation at time of review.
